# Liposomal delivery systems for intestinal lymphatic drug transport

**DOI:** 10.1186/s40824-016-0083-1

**Published:** 2016-11-23

**Authors:** Hyeji Ahn, Ji-Ho Park

**Affiliations:** Department of Bio and Brain Engineering, and Institute of Health Science and Technology, Korea Advanced Institute of Science and Technology (KAIST), 291 Daehak-ro, Yuseong-gu, Daejeon, 34141 Republic of Korea

**Keywords:** Chylomicron, First-pass metabolism, Intestinal lymphatic transport, Lipid, Liposome

## Abstract

Intestinal lymphatic drug delivery has been widely studied because drugs can bypass the first-pass metabolism in the liver via the lymphatic route, which increases oral bioavailability. Various lipid-based nanoparticles have been used to deliver hydrophobic drugs to the lymphatic pathway. This review focuses on the liposomal delivery systems used for intestinal lymphatic drug transport. Liposomal formulations have attracted particular attention because they can stimulate the production of chylomicrons and the incorporated drugs readily associate with enterocyte-derived chylomicrons, enhancing lymphatic drug transport. We believe that a full understanding of their contribution to intestinal drug translocation will lead to effective oral delivery with liposomal formulations.

## Background

**Fig. 1:**
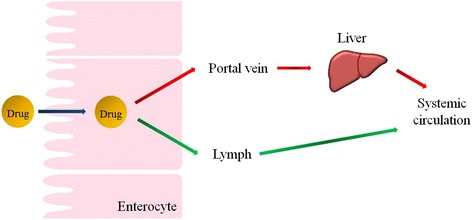
Two pathways (portal vein versus lymph) of oral drug transport to systemic circulation

There are many ways to deliver drugs into the body, including oral, pulmonary, subcutaneous, intravenous, transdermal, and nasal. Among them, oral drug delivery is particularly useful because it is convenient and comfortable for patients and thus is associated with a high rate of compliance. In other words, patients prefer drugs to be administered in oral form. However, orally administered drugs typically show low bioavailability due to their degradation by enzymes in the gastrointestinal (GI) tract, the difficulty of absorbing them in the small intestine, and the first-pass metabolism in the liver [1]. After oral administration, the drugs pass through the small intestine and enter the portal vein or intestinal lymphatic system (Fig. 1). Two main factors control the route that drugs take: molecular mass and solubility [2, 3]. Soluble, small drugs are preferentially transported via the portal vein. These drugs immediately accumulate in the liver and are then metabolized by enzymes, which lowers the drug concentration in the bloodstream. An alternative route for delivering drugs to the systemic circulation is the intestinal lymphatic pathway. Lipophilic drugs are known to be transported via the lymphatic system. The intestinal lymphatic pathway can bypass first-pass metabolism in the liver, thus increasing drug bioavailability. Furthermore, the co-administration of drugs with lipids can enhance their lymphatic transport [4]. In a postprandial state, lipid–drug conjugates and lipid-based nanoparticles have been widely studied for the delivery of lipophilic drugs via the lymphatic pathway. In this article, we highlight liposomal formulations developed to facilitate the lymphatic transport of loaded lipophilic drugs.

## Overview of intestinal lymphatic drug transport

### Assembly and secretion of chylomicrons

Chylomicrons are one group of lipoproteins. They consist of triglycerides (85–92%), phospholipids (6–12%), cholesterol (1–3%), and proteins (1–2%). Lipoproteins are macromolecules that transport hydrophobic lipids throughout the aqueous environment of the circulatory system [3, 5]. Orally administered exogenous lipids are absorbed into the apical side of enterocytes. Some monoglycerides and fatty acids diffuse into enterocytes and enter the portal vein, while others are resynthesized to chylomicrons and secreted into lymphatic vessels. In the process of chylomicron formation, monoglycerides and fatty acids are transformed to triglycerides by either the α-glycerol-3 phosphate pathway in the rough endoplasmic reticulum or the 2-monoglyceride pathway in the smooth endoplasmic reticulum [3, 6, 7]. Triglycerides synthesized by these two pathways then enter the endoplasmic reticulum lumen and are assembled into chylomicrons. These chylomicrons are transported to the Golgi and exocytosed from the enterocyte. Finally, the chylomicrons enter the lymphatic route and circulate throughout the body.

**Fig. 2:**
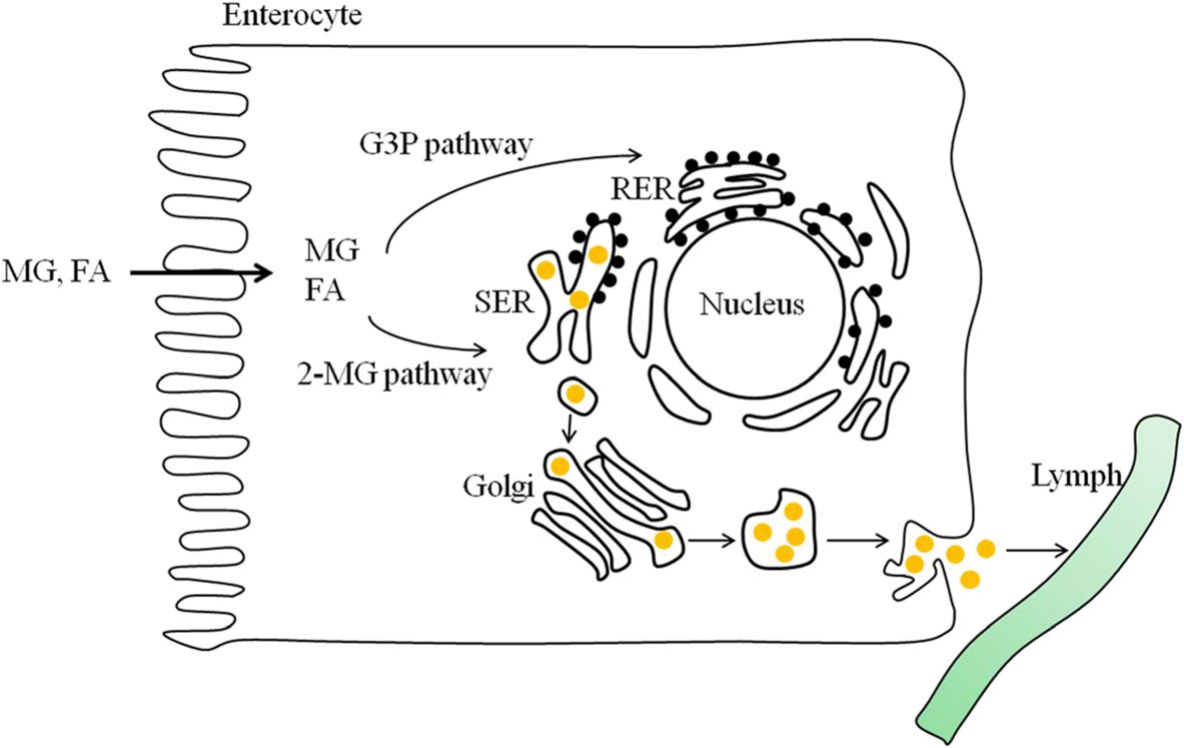
Pathways of lipid absorption and chylomicron synthesis within enterocytes

The lipid transport pathway varies depending on the lipid class, lipid-chain length, and degree of saturation (Fig. 2). Fatty acids with a chain length of 14 carbons or more are transported via the lymphatic pathway, while those with short chain lengths, which are more soluble, are transported via portal blood. Unsaturated fatty acids are also prone to be transported via the lymphatic pathway. Thus, these unsaturated and longer-chain fatty acids are assembled into larger lipoprotein chylomicrons and enter the intestinal lymphatic pathway [3, 8]. After the chylomicrons are exocytosed from the small intestine, they enter lymphatic vessels and transport dietary fats from the small intestine to peripheral tissues including muscle, adipose tissues, and heart. Triglycerides in chylomicrons are hydrolyzed by lipoprotein lipases to glycerol and fatty acids at the inner surface of capillaries in the peripheral tissues [9]. These chylomicron remnants are also taken up by liver cells by interacting with specific receptors in the liver.

### Incorporation of lipophilic drugs into chylomicrons for lymphatic transport

**Fig. 3:**
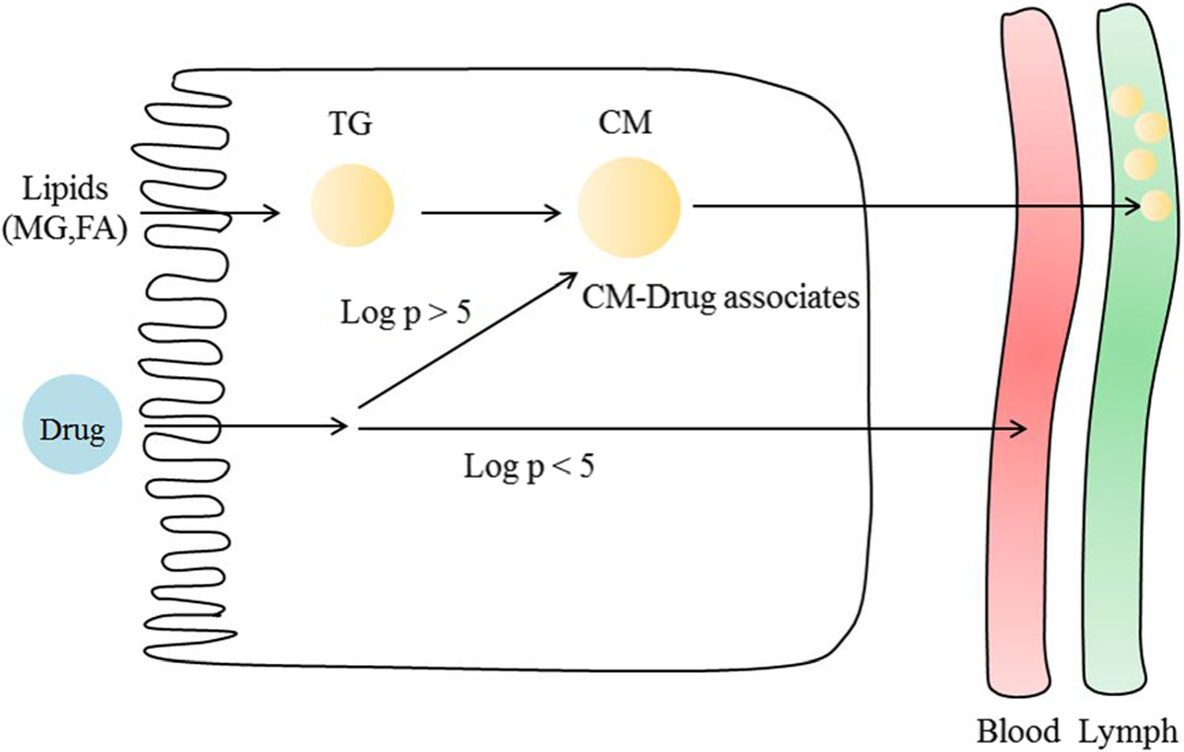
Lipophilic drug transport mechanisms by association with lipoproteins

Drugs can be transported into systemic circulation by either the portal vein or the lymphatic system. The partition coefficient (P) is the ratio of concentrations of a drug in two immiscible solutions, octanol and water. The log P value is a measure of drug lipophilicity. This value can thus be used to determine the transport pathway of drugs. Specifically, if the log P value of drugs is higher than 5, the drugs are highly lipophilic. It has been reported that lipophilic drugs associate with lipoproteins, specifically within their core [3, 6]. Lipoproteins associated with lipophilic drugs enter intestinal lymphatic vessels and are transported to systemic circulation. Co-administration with lipids can increase the level of drug transport through the intestinal lymphatic system (Fig. 3); thus, several approaches for the co-administration of lipophilic drugs with lipids have been studied (Table 1).

**Table 1:** Methods for incorporation of lipophilic drugs into chylomicrons

Method	Concept	Advantages	Disadvantages	References
Postprandial state (diet-derived)	Drugs are co-administered with food.	Chylomicrons are synthesized more than those of treating any other methods.	Uncontrollable method	[10] – [14]
Lipidic prodrugs	Drugs are covalently bound to a lipid moiety (fatty acid, glyceride, phospholipid).	Through covalent bonding with lipids, lipophilicity of drugs can be increased.	- Pharmaceutically inert - Safety and toxicity problems	[12] – [15]
Lipid-based nanoparticles (formulation-derived)	Administration of a single capsule of long-chain lipid can stimulate significant lymphatic transport of drugs. - Solid lipid nanoparticles - Self-nanoemulsifying drug delivery systems - Emulsion - Liposomes	- Effective carriers for drugs - Stimulators of chylomicron production	The effect of the formulation on drug absorption and lymphatic transport is less clear.	[13] – [18]

#### Postprandial state (diet-derived)

Chylomicron levels in the intestinal lymphatic duct increase substantially after a meal or feeding on fatty food. Fatty food enhances intestinal lipoprotein synthesis and the access of drugs to the intestinal lymphatic system. When drugs are co-administered with fatty food, their lymphatic transport increases dramatically. Shui-Mei Khoo et al. investigated the antimalarial drug halofantrine regarding intestinal lymphatic delivery [10]. The lymphatic transport of halofantrine accounted for 1.3% and 54% of the administered dose after fasting and in a postprandial state, respectively, suggesting that a postprandial state is the main factor influencing lymphatic halofantrine transport. More recently, Murakami et al. showed that, in a postprandial state, vitamin E–siRNA complex was administered by direct injection in a ligated loop of the large intestine and delivered delivered by the chylomicron-mediated lymphatic pathway [11]. Lipoprotein lipase changed the form from chylomicrons to chylomicron remnants, which finally accumulated in the liver. The siRNA delivered to the liver silenced apolipoprotein B gene expression.

#### Lipid-conjugated drugs

**Fig. 4:**
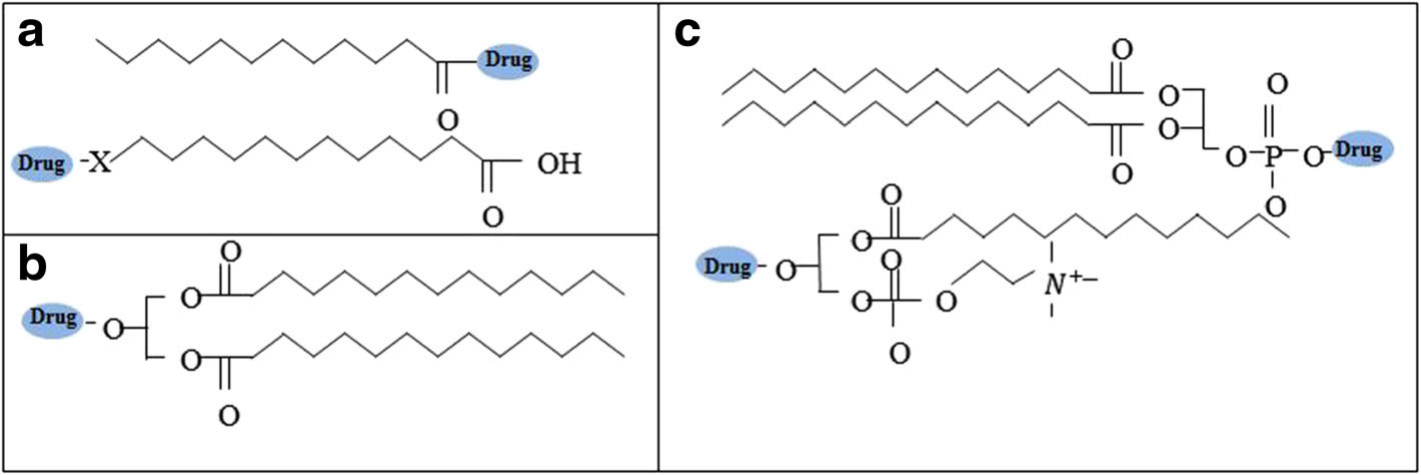
Types of lipidic prodrug: **a** fatty acids, **b** glycerides and **c** phospholipids

Drugs modified with a lipid moiety, such as a fatty acid, glyceride, or phospholipid, show increased lipophilicity (Fig. 4). These lipid-conjugated drugs can be associated into enterocyte-derived chylomicrons. However, such modified drugs should be considered as novel entities because this modification could raise problems of safety and toxicity. In addition, confirmation should be obtained that lipid-conjugated drugs show efficacy identical or similar to that of the original drug [1, 12, 13].

Gert Fricker et al. used a phospholipid–valproic acid conjugate as a model drug for oral delivery [13]. Phospholipid–drug conjugates have been shown to enhance the absorption and bioavailability of oral drugs. The absorption of this conjugate was examined in rats by administering long-chain or medium-chain triglyceride formulations. It was found that the bioavailability of the conjugates was enhanced when conjugates were administered with long-chain triglyceride formulations.

Drug lipophilicity increases by conjugation with the phospholipid, resulting in enhanced absorption into enterocytes. Arik Dahan et al. showed that phospholipid–drug conjugates have been associated with chylomicrons and reach the systemic circulation via the lymphatic pathway [15]. An example of using drug–glyceride conjugates to improve oral delivery has been also reported [16]. Specifically, mefenamic acid was modified with glyceride to reduce its side effects in the GI tract. The biolabile prodrugs of mefenamic acid showed higher plasma concentrations than upon treatment with the parent drug. These lipidic prodrugs thus increase bioavailability and reduce side effects in the GI tract.

#### Lipid-based nanoparticles (formulation-derived)

The administration of a single capsule of long-chain lipids can stimulate significant lymphatic transport of drugs. Exogenous lipids from various formulations enhance lymphatic transport by assembling into chylomicrons for endogenous lipid transport. Thus, drugs together with exogenous lipids can be delivered more efficiently to the intestinal lymphatic system through an increase in chylomicrons. Suzanne M. Caliph et al. showed lipid-based nanoparticles with long-chain lipids increased the oral bioavailability of lipophilic drugs [17]. Other studies reported that docetaxel nanocapsules consisting of long-chain triglycerides were transported in the form of lipoproteinated nanocapsules [18, 19]. These nanocapsules were recognized as triglyceride-rich particles in enterocytes, which resulted in them being transported through the intestinal lymphatic pathway. Chylomicron-mimicking carriers have also been developed to enhance the intestinal lymphatic pathway [20]. Carriers made of Compritol 888 ATO and soybean PC were recognized as chylomicrons in enterocytes. Methotrexate loaded in the carriers was delivered efficiently to the systemic circulation via the lymphatic route.

## Liposomal formulations for lymphatic drug transport

**Fig. 5:**
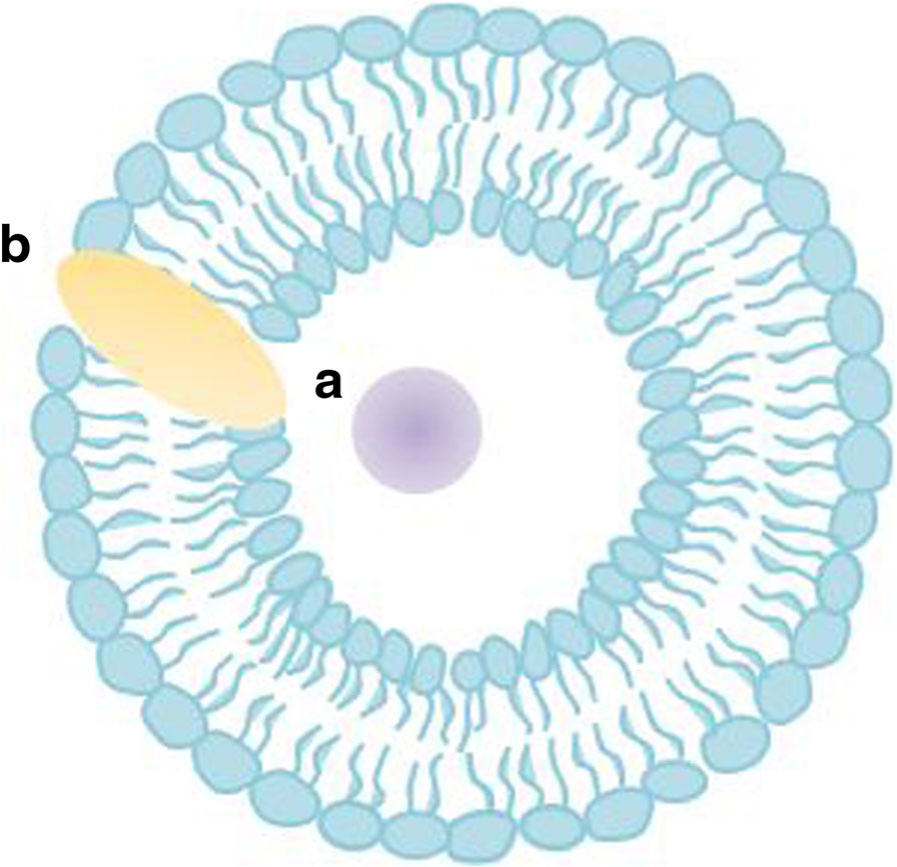
Liposomes with a hydrophilic drug **a** encapsulated in the aqueous core and a hydrophobic drug **b** incorporated into the membrane

Liposomes are spherical vesicles formed by one or several kinds of lipid with an aqueous phase inside and between the lipid bilayers [21–23]. Hydrophilic molecules can be loaded into the interior of liposomes, and hydrophobic or lipophilic molecules into the lipid bilayer (Fig. 5). Compared with other lipid-based nanoparticles, liposomes have the ability to encapsulate and protect drugs and to increase their absorption into enterocytes. Liposomes can protect labile drugs from denaturation by the harsh conditions in the GI tract. Lipids of liposomes can also be utilized to stimulate the production of chylomicrons in enterocytes, thus enhancing drug transport into the lymphatic system. Furthermore, enterocyte uptake of liposomes can be controlled with their size; smaller showed higher uptake [24]. Therefore, this section mainly focuses on liposomal formulations for oral drug delivery, especially lymphatic drug transport.

### Improvement of stability and bioavailability of drugs by liposomal formulations

Liposomal drugs have several advantages over free drugs for oral delivery. Liposomal formulations protect drugs from harsh conditions and slow down the rate of enzymatic degradation in the GI tract, thus improving drug stability and bioavailability [25, 26]. Kisel et al. showed that liposomes composed of phosphatidylethanol had capability to protect the incorporated drugs from pancreatic phospholipase [27]. Liposomal encapsulation also enhances the oral bioavailability of poorly absorbed hydrophilic drugs. Sharon Sheue Nee Ling et al. demonstrated increase in the oral bioavailability of cefotaxime, a hydrophilic drug, with liposomal formulations [28]. In addition, the liposomal formulation promoted lymphatic transport of cefotaxime. Cefotaxime concentrations in the lymph and plasma are higher when administered with liposomal formulations than when administered with an aqueous solution. These findings demonstrate that liposomal formulations can play an important role in improving drug stability and bioavailability for oral delivery, by facilitating lymphatic drug transport.

### Improvement of liposome absorption into enterocytes

Liposome formulations have been engineered by several methods to increase the efficiency of their uptake into enterocytes. First, surfactants such as bile salts can be incorporated into liposomes to enhance cellular uptake by disrupting the apical side of enterocytes [29, 30]. Bile salts incorporated into liposomes stabilize the liposomal membrane from bile acids in the GI tract. In addition, bile salt-incorporating liposomes show a prolonged residence time compared with conventional liposomes that incorporated cholesterol instead of bile salts. Second, elastic liposomes are taken up more efficiently by enterocytes [31]. Liposomes with Tween 80 and ethanol show elastic properties. These elastic liposomes exhibit good stability in simulated gastric and intestinal fluids, and sustained release of the incorporated drug. In oral delivery, elastic liposomes encapsulating (+)-catechin show an increased plasma level of (+)-catechin compared with free (+)-catechin. In addition, the modification of liposomes with Pluronic F127 can enhance their mucus penetration and cellular uptake [32]. Pluronic F127-incorporating liposomes also show more efficient delivery of coumarin 6 to enterocytes than unmodified liposomes.

### Effect of surface charge of liposomes on intestinal lymphatic drug transport

Modifying the surface charge of liposomal membranes increases cellular uptake and lymphatic drug transport. The residence time of liposomal formulations in the GI tract influences drug bioavailability. To increase the GI residence time, the surface charge of liposomes was engineered by coating them with carbopol or chitosan [33–35]. Chitosan and carbopol have mucoadhesive properties due to their positive charges. Therefore, a carbopol or chitosan coating improves the enteral absorption of liposomal drugs. In addition, in a recent study, liposomes were prepared with cationic stearylamine (SA), anionic phosphatidylserine (PS), or chitosan (CS) [36] and subjected to a mucoadhesion experiment. The liposomes prepared with SA showed better mucoadhesion than those prepared with PS and CS. This suggests that the positive charge of liposomes increases their mucoadhesion, resulting in enhanced intestinal lymphatic drug delivery.

## Conclusions

When drugs are orally administered, they can bypass first-pass metabolism through the lymphatic pathway. During intestinal lymphatic drug transport, long-chain and unsaturated lipids are assembled into chylomicrons in enterocytes. These chylomicrons are then exocytosed from the cell and enter the lymphatic route. If lipophilic drugs are co-administered with these lipids, they are prone to incorporation into chylomicrons and can be delivered to the lymphatic system in the form of chylomicron–drug complexes. Thus, co-administration with lipids can enhance the lymphatic transport of lipophilic drugs. There are several methods to enhance such lymphatic drug transport: administration during a postprandial state and the use of lipidic prodrugs and lipid-based nanoparticles. In particular, liposomes, which are the lipid-based nanoparticles most commonly used in drug delivery, have advantages over other methods in intestinal lymphatic drug transport because they can deliver various lipophilic drugs efficiently to enterocytes and utilize their phospholipids to stimulate the formation and exocytosis of chylomicron–drug complexes. Further research is needed to elucidate the effects of liposomal components on the formation and exocytosis of chylomicron–drug complexes in intestinal lymphatic delivery.
